# High-Intensity Focused Ultrasound in Aesthetic Plastic Surgery

**Published:** 2013-08-08

**Authors:** Kashyap K. Tadisina, Milan N. Patel, Karan Chopra

**Affiliations:** ^a^University of Illinois at Chicago College of Medicine; ^b^Department of Plastic and Reconstructive Surgery, The Johns Hopkins Hospital, Baltimore, Md

**Keywords:** body contouring, high intensity focused ultrasound, noninvasive body sculpting, aesthetic surgery

**Figure:**
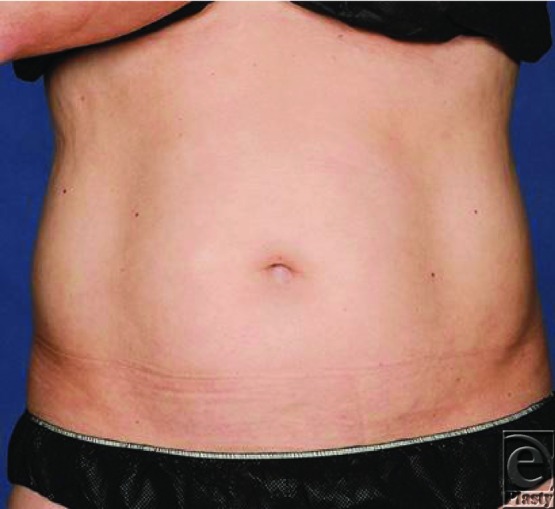


**Figure:**
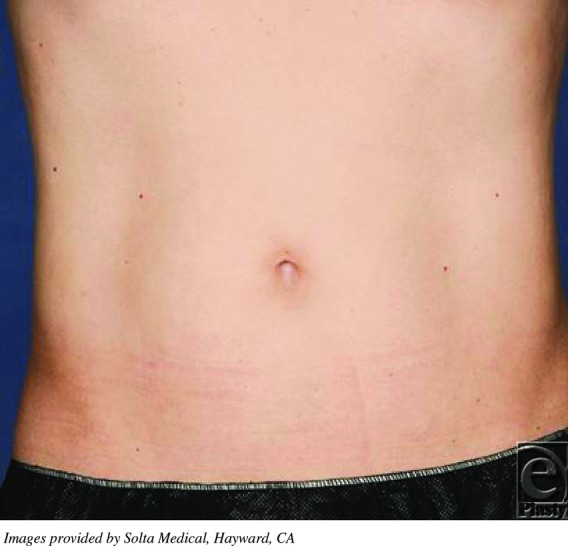


## DESCRIPTION

A 34-year-old healthy man presents requesting abdominal liposuction for excess abdominal fat. The patient had a strong preference for minimally invasive techniques. Therefore, High-intensity focused ultrasound (HIFU) therapy was performed.

## QUESTIONS

1. **What is HIFU and how does it work?**
2. **What is HIFU currently used for?**
3. **What are some of the recognized shortcomings and complications of HIFU?**
4. **What is the future of HIFU?**

## DISCUSSION

High-intensity focused ultrasound is the result of the evolution of ultrasound from a simple diagnostic procedure to a therapeutic modality with broader potential. Ultrasound has long been an intriguing medical modality because of its noninvasive nature, low cost, and relatively low rate of complications.^1^ Sound is transmitted by mechanical vibrations; ultrasound technology utilizes vibrations that are outside of the range of human hearing. High-intensity focused ultrasound, in comparison to typical ultrasound, uses lower frequencies and higher energy levels by several orders of magnitude.^1^ As compared to normal ultrasound, where acoustic waves are resorbed and deflected through tissue, HIFU can target a specific volume (on the order of millimeters) within the body cavity without harming surrounding tissues.^2^ A major advantage is that the energy is nonionizing and can theoretically be repeated an unlimited number of times. High-intensity focused ultrasound works via 2 major underlying mechanisms, the first of which is hyperthermia. Concentrated energy causes temperatures to exceed the upper limit of protein denaturation (43° C), and can reach as high as 80° C, causing instant coagulative necrosis of targeted cells without damage to surrounding areas.^3^ The second major mechanism is cavitation formation. At higher energy levels, alternating compression and expansion of sound waves create gas cavities that implode and subsequently cause mechanical damage to the target tissues (eg, adipose) through the release of high levels of pressure and heat in the microenvironment.^2^ This mechanism, although more powerful, tends to be more unpredictable and harder to control, and thus its use is limited compared to hyperthermia.

High-intensity focused ultrasound is most widely recognized in the treatment of benign and malignant solid tumors.^4^ It has been successfully used to treat tumors of the liver, kidneys, pancreas, breast, prostate, and even bone.^4^ Most recently, HIFU has been implemented in the outpatient setting as a technology used for cosmetic procedures. Although radiologic techniques have been used as an adjunct to traditional liposuction in the past, this is the first ultrasound technique used as the primary form of adipocyte reduction.^5^ High-intensity focused ultrasound has been used effectively for noninvasive body sculpting to either tighten skin by contracting collagen fibers or remove adipose tissue stores via ablation.^6^ Previous studies have demonstrated that optimal results are obtained when the patients have a BMI less than 30 kg/m^2^ and have at least 1 cm of adipose tissue beyond the treatment area.^7^^,^^8^ Average treatment times are reported at 45 to 60 minutes with minimal to no recovery time. Patient satisfaction rates have been reported as high as 70% at 3 months follow-up.^9^ Fatemi et al have shown that within 18 weeks of HIFU-mediated adipose tissue ablation, 95% of the cellular debris had been reabsorbed, without posttreatment changes in patients' lipid profiles or comprehensive metabolic panels.^8^^,^^9^ It has also been employed as an adjunct to craniofacial surgical procedures, namely to minimize postrhinoplasty nasal tip edema.^10^ Utilizing thermal coagulation to cause contraction of collagen fibers, skin was “drawn taught” over underlying collagenous architecture. Concurrently, small amounts of adipose tissue can be removed, making this a useful treatment option in patients who have not had success with other treatment modalities due to either their skin or anatomical contours.^11^

Traditional shortcomings of HIFU include those of diagnostic ultrasound, most importantly that sound waves cannot pass through solid structures (ie, bone) and are impeded by air, limiting its use in hollow organs such as the lungs.^4^ Furthermore, anesthesia is required for longer and more complicated anatomical procedures, particularly in enlarged tumor ablation. When utilizing ultrasound, it is vital to know patients' individual anatomy, as a proper “acoustic” window must be available in order for sound waves to reach their desired location. If solid objects such as organs, prostheses, or bones lie in between the target and transducer, other modalities must be considered as HIFU's efficacy is dramatically reduced.^1^ In the setting of noninvasive body sculpting, patients have reported feeling localized tingling/prickling sensations along with mild warmth and pain during treatment. Commonly reported posttreatment adverse effects include discomfort, ecchymosis, paresthesias, and edema. All of these are temporary and mild in intensity, with the vast majority resolving within 12 weeks.^6^^,^^12^

High-intensity focused ultrasound has only recently made its debut in the plastic surgery community, with preliminary studies being promising and exciting. It has been shown to be safe at much higher doses and duration than are typically necessary in the outpatient setting^6^ with initial studies showing high patient satisfaction and reproducible results,^3^ making it an option for plastic surgeons to consider when encountering patients requesting body contouring or liposuction, particularly those who are poor candidates for or resistant to surgery. Research is also being conducted evaluating the utilization in nontraditional settings such as thrombolysis, vascular hemostasis, drug/gene therapy,^4^ and targeted tumor cell chemotherapy.^13^ Advancements in the intraoperative assessment of temperature with magnetic resonance imaging,^2^ localization with 3-dimensional ultrasound,^2^ and quantification of collagen contraction with elastography^1^ combined with the expected advancements in ultrasound technology pave the way for HIFU to potentially revolutionize the future of noninvasive plastic surgery. As medicine moves toward less invasive modalities, such as interventional radiology and laparoscopic and robotic procedures, HIFU is poised to meet the demand of many clinical scenarios including solid tumor treatment, vascular injury, drug delivery, and both aesthetic and reconstructive procedures.
